# Comparison of DNA Extraction Protocols and Molecular Targets to Diagnose Tuberculous Meningitis

**DOI:** 10.1155/2017/5089046

**Published:** 2017-05-30

**Authors:** Flavia Silva Palomo, Martha Gabriela Celle Rivero, Milene Gonçalves Quiles, Fernando Pereira Pinto, Antonia Maria de Oliveira Machado, Antonio Carlos Campos Pignatari

**Affiliations:** ^1^Special Clinical Microbiology Laboratory (LEMC), Federal University of São Paulo (UNIFESP), São Paulo, SP, Brazil; ^2^Central Laboratory of São Paulo Hospital, Federal University of São Paulo (UNIFESP), São Paulo, SP, Brazil

## Abstract

Tuberculous meningitis (TBM) is a severe form of extrapulmonary tuberculosis. The aims of this study were to evaluate in-house molecular diagnostic protocols of DNA extraction directly from CSF samples and the targets amplified by qPCR as an accurate and fast diagnosis of TBM. One hundred CSF samples from 68 patients suspected of TBM were studied. Four DNA extraction techniques (phenol-chloroform-thiocyanate guanidine, silica thiocyanate guanidine, resin, and resin with ethanol) were compared and CSF samples were used to determine the best target (*IS6110*,* MPB64,* and* hsp65 KDa*) by qPCR. The extraction protocol using the phenol-chloroform-thiocyanate guanidine showed the best results in terms of quantification and sensitivity of PCR amplification, presenting up to 10 times more DNA than the second best protocol, the silica guanidine thiocyanate. The target that showed the best result for TBM diagnosis was the* IS6110*. This target showed 91% sensitivity and 97% specificity when we analyzed the results by sample and showed 100% sensitivity and 98% specificity when we analyzed the results by patient. The DNA extraction with phenol-chloroform-thiocyanate guanidine followed by* IS6110 target* amplification has been shown to be suitable for diagnosis of TBM in our clinical setting.

## 1. Background

Tuberculosis is a serious infectious contagious disease that usually affects the lungs but can also affect other organs such as kidney, bone, and central nervous system (CNS) [1]. In 2011, cases of extra pulmonary tuberculosis in Brazil reached almost 16% of all cases of the disease [2] and about 6.3% of these (1.3% of the total) were TBM [3].

The TBM is the most severe form of extra pulmonary tuberculosis that has a high morbidity and mortality [4, 5]. The definitive diagnosis of TBM depends on the* M. tuberculosis* agent detection from cerebrospinal fluid (CSF). Routinely, the survey of* M. tuberculosis* in CSF is carried out by conventional microbiological methods including Ziehl-Neelsen smear, which has low sensitivity (0–20%) and culture, which requires until 65 days to final result [1, 6].

The sensibility for detection of* M. tuberculosis* in CSF samples can be substantially increased from 70% to 100% and the time required to release laboratory results can be significantly decreased with the use molecular methods, including the polymerase chain reaction (PCR). The rapid identification of TBM through molecular analysis of CSF is an important factor for proper and early institution of antimicrobial treatment [7–9].

The aims of this study were to evaluate in-house molecular diagnostic protocols of DNA extraction directly from CSF samples and the targets amplified by qPCR as an accurate and fast diagnosis of TBM.

## 2. Methods

### 2.1. Clinical CSF Samples

We utilized the CSF samples sent for mycobacteria culture at the Central Laboratory of São Paulo Hospital, Federal University of São Paulo/UNIFESP, Brazil, in the period from January 2011 to June 2014. Aliquots were frozen at −20°C and submitted to molecular tests after being thawed and centrifuged. These samples were also subjected to biochemical and cytological analysis and determination of adenosine deaminase (ADA) levels.

The samples were classified as true positive and negative after a survey in the database “BrazilianTBweb”, other laboratory CSF data and evaluation of medical records.

### 2.2. Microbiological Diagnostic of CSF Samples

The microbial diagnosis of CSF samples was made in the Central Laboratory of São Paulo Hospital, Federal University of São Paulo/UNIFESP, Brazil. For each CSF a Ziehl-Neelsen smear and mycobacterium culture in Lowenstein Jensen solid medium were done.

### 2.3. DNA Extraction Protocols

Four extraction methods were analyzed: phenol-chloroform with guanidine thiocyanate (Brazol®, LabTrade, Brazil), silica-guanidine plus thiocyanate plus guanidine thiocyanate (QIAmp® DNA Mini Kit, Qiagen, USA), resin (Chelex® 100 resin, BioRad, USA), and resin precipitated with ethanol.

Comparison of extraction was determined by a serial 10-fold dilutions prepared in four different diluents (ultrapure water, turbid, xanthochromic, and hemorrhagic CSF pools known negative for TBM) from a 0.5 McFarland (1.5 × 10^8^ CFU/mL) suspension of* M. tuberculosis* ATCC 25177.

The extracted DNA was measured and analyzed by NanoVue ND-100 (*Thermo Fisher Scientific*, Wilmington, USA) and the amplification sensitivity was determined by* IS6110* target. The DNA extraction protocol presenting the best results was chosen for target and clinical samples analysis.

*Phenol-Chloroform-Thiocyanate Guanidine (Brazol, LabTrade, Brazil) DNA Extraction*. An aliquot of 200 *μ*L CSF sample sediment was added to a microtube containing 500 *μ*L of Brazol and mixture. Then ice-cold chloroform (300 *μ*L) was added. The mixture was homogenized and centrifuged at 12,000 rpm for 15 minutes at 8°C. The supernatant was removed and transferred to another microtube containing 500 *μ*L of cold absolute ethanol. The mixture was stirred for 3–5 seconds by vortexing and centrifuged at 12,000 rpm for 15 minutes at 8°C. The supernatant was removed and the “pellet” was washed with 500 *μ*L of ice-cold ethanol and centrifuged at 12,000 rpm for 15 minutes at 8°C. The supernatant was carefully removed and discarded. The “pellet” was incubated at room temperature to dryness and then dissolved in 30 *μ*L of ultrapure water (Invitrogen Life Technologies, USA) and allowed to dissolve at 65°C for 30 minutes. The samples were frozen at −20°C when not used immediately.

*Silica-Guanidine Thiocyanate (QIAamp DNA Mini Kit, Qiagen, USA) DNA Extraction*. The protocol recommended by the manufacturer was used with 200 *μ*L of the CSF sediment.

*Resin (Resin Chelex100, BioRad, USA) DNA Extraction*. A 200 *μ*L aliquot of the CSF sample pellet was placed in 300 *μ*L of a 10% solution Chelex. This mixture was homogenized in vortex for 10 seconds, centrifuged quickly to remove excess fluid from the cover, and incubated at 95°C for 30 minutes in a heat block. The tube was homogenized for 15 sec and centrifuged for 5 minutes at 13000 rpm. The supernatant containing the extracted DNA was transferred to a new tube. When not used immediately, the sample was frozen at −20°C.

*Resin Precipitated with Ethanol (Chelex100 Resin, BioRad, USA) DNA Extraction*. A 200 *μ*L aliquot of the CSF pellet was placed in 300 *μ*L of 10% solution Chelex. This mixture was homogenized in vortex for 10 seconds and centrifuged quickly to remove excess fluid from the cover. This mixture was incubated at 95°C for 30 minutes in a heat block. The tube was homogenized for 15 sec and centrifuged for 5 minutes at 13000 rpm. The supernatant containing the extracted DNA was transferred to a new tube and 500 *μ*L of cold absolute ethanol was added. The mixture was stirred for 3–5 seconds by vortexing and centrifuged at 12,000 rpm for 15 minutes at 8°C. The “pellet” was washed with 500 *μ*L of ice-cold ethanol and centrifuged at 12,000 rpm for 15 minutes at 8°C. The supernatant was carefully removed and discarded. The “pellet” was incubated at room temperature to dryness and then dissolved in 30 *μ*L of ultrapure water (Invitrogen Life Technologies, USA) and allowed to dissolve at 65°C for 30 minutes. The samples were frozen at −20°C when not utilized immediately.

### 2.4. Targets and PCR Protocols

Three different targets were analyzed for* M. tuberculosis* detection by real time PCR:* IS6110* gene, 65 kDa Heat Shock Protein gene* (hsp65 KDa),* and the MPB64 protein encoding gene* (MPB64)* (Table 1). The reaction was performed on Rotor-Gene 5 plex/HRM platform (Qiagen, USA) by melting analyses (QuantiFast SYBR Green PCR (Qiagen, USA)) on 20 *μ*L final volume. The limit of detection (LoD) was determined by a serial sevenfold dilutions prepared in ultrapure water (Sigma-Aldrich, Co. Ltd, Saint Louis, US).

The target specificity was carried out by testing the negative control group and CSF samples. The negative control group was made up by ATCC and known strains of bacteria, yeasts, and other mycobacteria (Table 2).

### 2.5. Real Time PCR Protocol

A reaction containing 10 *μ*L of QuantiFast SYBR Green PCR (Qiagen, USA), 0.5 mM of each primer, and 2 *μ*L of sample was used for* M. tuberculosis* DNA detection directly from the CSF sample. Thermocycling was performed in the PCR system in the real time Rotor-Gene Q 5plex Platform (Qiagen, USA) using the following conditions: an initial cycle of five minutes at 95°C, followed by 45 cycles of 30 seconds at 94°C and 45 seconds at the respective annealing temperature for IS6110 (65°C), hsp65 KDa (62°C), and MPB64 (61°C). At the conclusion of cycling a melting step ranging from 72°C to 95°C with an increase of 0.5°C per second was added for each gene.

### 2.6. Statistical Analysis

The results obtained were compared and evaluated clinically and analytically. For each test sensitivity (*S*), specificity (SP), positive predictive value (PPV), negative predictive value (NPV) and accuracy (*A*) were calculated. The* Kappa* index (*k*) was used to analyze the correlation between the tests. For analytical comparison, mycobacteria culture was considered as gold standard. For clinical analysis the patients and their respective samples were classified as true positive and true negative in accordance with the result of mycobacterium culture and clinical and laboratory data. Clinical data of patients were collected from electronic medical records and laboratory reports provided by the Central Laboratory of Hospital São Paulo and records in Brazilian TBweb (http://www.cvetb.saude.sp.gov.br/).

## 3. Results

Among 100 clinical CSF samples collected from 68 patients, 35 from 16 patients were considered true positive and 65 samples from 52 patients were true negative by clinical parameters.

The DNA extraction protocol using the phenol-chloroform-thiocyanate guanidine presented the highest final DNA concentration among four methods evaluated (Table 3).

From 35 clinical samples considered true positive by clinical parameters for TBM diagnosis, IS6110 PCR was able to detect 32 of them followed by culture and* hsp65 KDa* (16 samples) and* MPB64 * (12 samples). (Table 4).

Sixty-five samples were considered as true negative by clinical parameters for TBM diagnosis and culture was negative for all of them while 63 samples were negative by* IS6110*,* MBP64,* and* hsp65 KDa* PCR. (Table 4).

From 16 patients considered true positive by clinical parameters for TBM diagnosis,* IS6110* was able to correctly detect all of them followed by* hsp65 KDa* (11 patients), culture (10 patients), and* MBP64 *(7 samples). (Table 5).

From 52 patients considered true negative by clinical parameters for TBM diagnosis, the culture was able to detect all of them as negative followed by IS6110 (51 patients), hsp65, and MBP64 (50 samples). (Table 5).

LoD, efficiency, Ct (cycle threshold) median and *T*_m_ (temperature of melting) median for* IS6110*, MPB* 64, *and hsp65 KDa are presented in Table 6. The primers were specific for* M. tuberculosis *and did not show cross reactivity against different microorganisms tested.

## 4. Discussion

Over the years several studies proposed and validated molecular techniques for diagnosis of TBM [8–14]. These studies considered the importance of developing a simple technique that was easily reproduced in laboratories with minimal resources [10, 11, 13, 15, 16]. The choice of the most appropriate extraction method and the target to be amplified are criteria that improve the precision, sensitivity and specificity of the PCR test [16–18].

The complexity of rich cell wall lipids of mycobacteria is a limiting factor for the success of some DNA extraction techniques [19–22], besides the fact that the microorganism is an intracellular pathogen, which can hinder the isolation of these microorganisms in clinical samples [19, 22]. Since the CSF samples from patients with TBM are usually paucibacillary it is recommended to recover the greatest amount of DNA as possible.

The extraction protocol using the phenol-chloroform-thiocyanate guanidine showed the best results in terms of quantification and sensitivity of PCR amplification, presenting up to 10 times more DNA than the second best protocol, the silica guanidine thiocyanate.

The techniques using phenol-chloroform, silica, and thiocyanate guanidine are described as excellent choices for DNA extraction in different biological materials, in addition to being inexpensive and simple [8–13].

The methodology of phenol-chloroform extraction and DNA purification utilized the Brazol, which has in its composition in addition to phenol, guanidine thiocyanate, a chaotropic agent that inactivates endonucleases and prevents DNA binding to other molecules and facilitates the separation of cellular debris [23–26]. Furthermore, the phenol which is a potent proteolytic agent, corrosive and caustic, contributes to lysis of the cell envelope of mycobacteria. In phenol extraction, solubilization and denaturation of proteins and lipids occur efficiently [26]. The chloroform used in this method is also a good protein denaturing detergent and a major solvent of fats, which probably contributed to the removal of the lipid layer of the mycobacteria cell wall. Regarding the biohazard risk of phenol-chloroform, all tests were done following the biosafety manuals and chemical waste disposal regulations of our country.

The target that showed the best amplification results was the* IS6110* qPCR with a sensitivity of 100% and specificity of 79%, when compared to culture. The sample analysis for* IS6110* qPCR amplification showed 91% sensitivity and 97% specificity with the clinical diagnosis. When this analysis was grouped by patient, we showed a very good agreement with the clinical diagnosis with 100% sensitivity and 98% specificity.

These results can be explained since the gene encoding the* MPB64* protein and the encoding gene* heat shock protein* (*hsp*65 KDa) have single copies in mycobacteria genome while the insertion gene* IS6110* has multiple copies which makes the reaction more sensitive [7–9].

The* IS6110* target sequence is a repetitive insertion of 1,350 base pairs present in* M*.* tuberculosis* complex species with different numbers of copies integrated into various chromosomal sites [9, 27, 28]. This molecular target promotes an increase in the sensitivity of the technique, which is an advantage over other targets. Only three patients had positive CSF samples for target* IS6110* undiagnosed for TBM, but when we evaluated other laboratory criteria or clinical characteristics of these patients two of them had TBM and only one remained doubtful.

Commercial molecular tests for pulmonary tuberculosis diagnosis such as XpertMTB/RIF represent a significant advance, since it is automated and provides fast results. However, for TBM diagnosis its sensitivity is around 60% to 80%. [29–31]. Other targets also can be used for* M. tuberculosis* molecular diagnosis, including the TRC4 primer a conserved repetitive element with specificity for* M. tuberculosis *complex [32].

There are few reports of* M. tuberculosis* lacking the* IS6110* that eventually could be responsible for false negative results [32, 33]; however, we considered that the benefit of a greater sensitivity of the* IS6110* gene that can be found repeatedly in the genome* M. tuberculosis* justifies its use [11, 32, 33].

Thus, we believe we have demonstrated the feasibility of a molecular test for the diagnosis of TBM by an in-house real time PCR with analytical and clinical correlation to be used in laboratories with adequate cost benefit. Studies comparing other molecular methods of DNA extraction, other molecular targets, in-house protocols, and commercial platforms are warranted.

## 5. Conclusion

The combination of DNA extraction by phenol-chloroform and guanidine thiocyanate, (Brazol) and qPCR by* IS6110* target amplification could be an effective tool for* M. tuberculosis *diagnosis directly from CSF samples.

## Figures and Tables

**Table 1:** Real time PCR targets primers.

Target	Primer	Sequence (5′-3′)	Product	Reference
*IS6110*	*IS-Fw*	CGTGAGGGCATCGAGGTGGC	245 bp	[9]
	*IS-Rv*	CGCTAGGCGTCGGTGACAAA		
*Hsp 65 KDa*	*hsp65-Fw*	GAGATCGAGCTGGAGGATCC	383 bp	[7]
	*hsp65-Rv*	AGCTGCAGCCCAAAGGTGTT		
*MPB64*	MPB64-Fw	TCCGCTGCCAGTCGTCTTCC	240 bp	[7]
	MPB64-Rv	GTCCTCGCAGTCTAGGCCA		

*Note*. Fw: forward; Rv: reverse; bp: base pairs of nucleotidis.

**Table 2:** List of microorganisms used as negative control.

Microorganisms	Origin
*Acinetobacter baumannii*	ATCC 19606
*Candida *spp.	Known strain
*Cryptococcus neoformans*	Known strain
*Enterococcus faecalis*	ATCC 29212
*Enterococcus faecium*	Known strain
*Escherichia coli*	ATCC 35218
*Haemophilus influenzae*	Known strain
*Haemophilus *spp.	Known strain
*Klebsiella pneumoniae *	Known strain
*Listeria monocytogenes*	Known strain
*Mycobacterium chelonae*	Known strain
*Mycobacterium abscessus*	Known strain
*Mycobacterium avium*	Known strain
*Mycobacterium gordonae*	Known strain
*Mycobacterium intracellulare*	Known strain
*Mycobacterium kansasii*	Known strain
*Mycobacterium lentiflavum*	Known strain
*Neisseria meningitidis*	Known strain
*Neisseria *spp.	Known strain
*Nocardia *spp.	Known strain
*Proteus mirabilis*	ATCC 29245
*Pseudomonas aeruginosa*	ATCC 27853
*Salmonella enterica*	ATCC 14028
*Shigella flexneri*	ATCC 12022
*Staphylococcus aureus*	ATCC 29213
*Staphylococcus epidermidis*	ATCC 12228
*Staphylococcus saprophyticus*	ATCC 43867
*Streptococcus agalactiae*	ATCC 12386
*Streptococcus pneumoniae*	ATCC 49619
*Streptococcus pyogenes*	ATCC 19615

ATCC: American Type Collection Culture.

**Table 3:** Nanovue results form four extraction protocols.

Diluent	Extraction method	Sensitivity amplification	DNA [ng/*μ*L]	A260/280
Ultrapure water	Phenol Chloroform	*10* ^*-10*^	*24,5*	*1,53*
	Silica	10^−9^	2,3	1,18
	Resin	10^−7^	1,2	0,93
	Resin ethanol	10^−4^	0,3	0,36

Turbid CSF	Phenol Chloroform	*10* ^*-10*^	*28,5*	*1,59*
	Silica	*10* ^*-10*^	22,2	1,45
	Resin	10^−7^	19	1,08
	Resin ethanol	10^−3^	0,1	0,01

Xanthochromic CSF	Phenol Chloroform	*10* ^*-10*^	*53*	1,56
	Silica	10^−9^	21	*1,8*
	Resin	10^−7^	24,5	1,09
	Resin ethanol	10^−6^	0,12	1,4

Hemorrhagic CSF	Phenol Chloroform	*10* ^*-10*^	*64,5*	1,55
	Silica	*10* ^*-10*^	60,8	*1,76*
	Resin	10^−7^	67	1,22
	Resin ethanol	10^−6^	0,1	2,19

**Table 4:** Culture and real time PCR results for 100 samples included on the study.

	*S*	SP	PPV	NPV	*A*	*K*
Culture	46%	100%	100%	77%	81%	0,52 (Weak)
*INS6110 *	*91%*	*97%*	*94%*	*95%*	*95%*	*0,89 (Strong)*
*MPB64 *	34%	97%	86%	73%	75%	0,36 (Minimal)
*hsp65 KDa *	46%	97%	89%	77%	79%	0,48 (Weak)

*Note*. *S*: sensitivity; SP: specificity; PPV: positive predictive value; NPV: negative predictive value; *A*: accuracy; *k*: Kappa index.

**Table 5:** Culture and real time PCR results for 100 samples included on the study.

	*S*	SP	PPV	NPV	*A*	*K*
Culture	63%	*100%*	*100%*	90%	91%	0,72 (moderate)
*INS6110 *	*100%*	98%	94%	*100%*	*99%*	*0,96 (almost perfect)*
*MPB64 *	44%	96%	78%	85%	84%	0,47 (weak)
*hsp65 KDa *	69%	96%	85%	91%	90%	0,69 (moderate)

*Note*. *S*: sensitivity; SP: specificity; PPV: positive predictive value; NPV: negative predictive value; *A*: accuracy; *k*: Kappa index.

**Table 6:** LoD, efficiency, Ct, and Tm results for real time PCR.

Target	LoD (CFU)	Efficiency	Ct	*T*_m_
			Median	Median
*IS6110*	10^0^	1,07	31,09	89,8
*Hsp 65 KDa*	10^2^	1,02	25,78	86,8
*MPB64*	10^3^	1,07	35,07	90,7

LoD: limit of detection; Ct: cycle threshold; *T*_m_: temperature of melting.
